# Di-*tert*-butyl cyclo­hex-2-ene-1,4-diyl dicarbonate

**DOI:** 10.1107/S1600536809040343

**Published:** 2009-10-10

**Authors:** Syed Nawazish Ali, Mitchell A. Winnik, Sabira Begum, Alan J. Lough

**Affiliations:** a H.E.J. Research Institute of Chemistry, International Center for Chemical and Biological Sciences, University of Karachi, Karachi 75270, Pakistan; b Department of Chemistry, University of Toronto, Toronto, Ontario, Canada M5S 3H6

## Abstract

In the title mol­ecule, C_16_H_26_O_6_, the central cyclo­hexene ring is in a half-chair conformation. The carbonyl groups are in a *trans* arrangement with respect to each other and the dihedral angle between the mean planes of the carbonate groups is 10.8 (2)°.

## Related literature

For synthetic applications of the title compound, see: Ali, Ghafouri *et al.* (2008 ▶). For a related structures, see: Ali, Begum *et al.* (2008 ▶); Rademeyer *et al.* (2003 ▶).
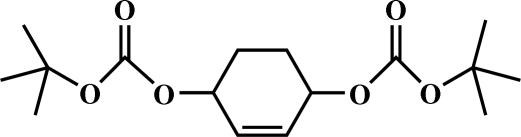


## Experimental

### 

#### Crystal data

C_16_H_26_O_6_
*M* *_r_* = 314.37
Monoclinic, 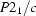

*a* = 12.6548 (11) Å
*b* = 5.8862 (6) Å
*c* = 23.126 (2) Å
β = 103.147 (5)°
*V* = 1677.5 (3) Å^3^
*Z* = 4
Mo *K*α radiation
μ = 0.09 mm^−1^
*T* = 150 K
0.10 × 0.09 × 0.02 mm

#### Data collection

Nonius KappaCCD diffractometer
Absorption correction: multi-scan (*SORTAV*; Blessing 1995 ▶) *T*_min_ = 0.865, *T*_max_ = 1.00
9313 measured reflections
2893 independent reflections
1407 reflections with *I* > 2σ(*I*)
*R*_int_ = 0.101

#### Refinement

*R*[*F* ^2^ > 2σ(*F* ^2^)] = 0.068
*wR*(*F* ^2^) = 0.191
*S* = 1.00
2893 reflections
205 parameters
H-atom parameters constrained
Δρ_max_ = 0.24 e Å^−3^
Δρ_min_ = −0.26 e Å^−3^

### 

Data collection: *COLLECT* (Nonius, 2002 ▶); cell refinement: *DENZO-SMN* (Otwinowski & Minor, 1997 ▶); data reduction: *DENZO-SMN*; program(s) used to solve structure: *SIR92* (Altomare *et al.*, 1994 ▶); program(s) used to refine structure: *SHELXTL* (Sheldrick, 2008 ▶); molecular graphics: *PLATON* (Spek, 2009 ▶); software used to prepare material for publication: *SHELXTL*.

## Supplementary Material

Crystal structure: contains datablocks global, I. DOI: 10.1107/S1600536809040343/ez2186sup1.cif

Structure factors: contains datablocks I. DOI: 10.1107/S1600536809040343/ez2186Isup2.hkl

Additional supplementary materials:  crystallographic information; 3D view; checkCIF report

## Figures and Tables

**Fig. 1.**
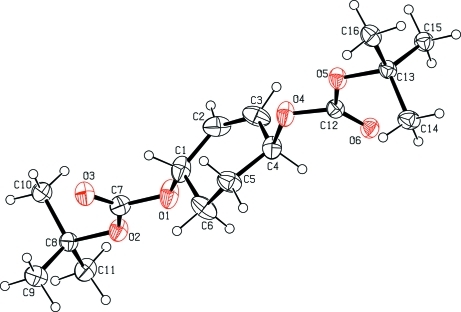
The molecular structure of the title compound showing 30% probability ellipsoids.

**Fig. 2.**
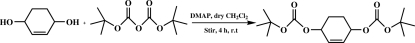
Preparation of the title compound.

## supplementary crystallographic information

### Comment

The title compound (I), is a new synthetic precursor of *trans*-cyclohex-2-ene-1,4-diol which has been synthesized for plasticizing purposes in order to break the crystallinity of a number of polyformals, and polycarbonates (Ali, Ghafouri *et al.*, 2008). The molecular structure of (I) is shown in Fig. 1. Unlike the crystal structure of *trans*-Cyclohex-2-ene-1,4-diyl bis(4-nitrophenyl) dicarbonate (Ali, Begum *et al.*, 2008) the central cyclohexene ring is completely ordered.

### Experimental

A reaction mixture containing *trans*-cyclohex-2-ene-1,4-diol (0.59 g, 5.18 mmol), di-*tert*-butyldicarbonate (2.26 g, 10.36 mmol) and *N*,*N*-dimethylaminopyridine (DMAP) (0.80 g, 6.57 mmol) was stirred in dry dichloromethane (80 ml) at room temperature in a 250 ml round-bottom flask (see Fig. 2). After 4 h, it was transferred to a separatory funnel (250 ml) and washed with CH_3_COOH (30 ml *x* 3, 0.1 *M*) to remove the excess of DMAP. The lower organic phase was removed and the aqueous phase was washed with dichloromethane (30 ml *x* 2). All the dichloromethane solutions were combined, washed with deionized water (30 ml *x* 3), and dried over anhydrous MgSO_4_. After filtration, the solvent was removed by rotary evaporator. The resulting oily product was dried in vacuum oven at room temperature to obtain di-*tert*-butyl-cyclohex-2-ene-1,4-diyl dicarbonate (I, 1.14 g, 69.5%). The product was then recrystallized from a mixture of CHCl_3_: MeOH (1:1) to afford needle-shaped crystals by slow evaporation of the solvent at room temperature. In addition to the X-ray structure determination, the structure was also confirmed by comparing the ^1^H and ^13^C NMR data with a related t-Boc protected compound (Rademeyer *et al.*, 2003). ^1^H NMR (CDCl_3_, p.p.m., relative to TMS, 400 MHz): 5.98 (2*H*, br.s, CH=CH), 5.16 (2*H*, m, CH—O), 2.08 (2*H*, m, CH_2_—CH_2_), 1.80 (2*H*, m, CH_2_—CH_2_), 1.48 (18*H*, s, CH_3_); ^13^C NMR (CDCl_3_, p.p.m., relative to TMS, 100 MHz): 168.4 (C=O), 129.1 (CH=CH), 71.4 (2*H*, CH—O), 66.3 (C(CH_3_)_3_O), 25.2 (CH_2_), 28.0 (CH_3_)

### Refinement

Hydrogen atoms were placed in calculated positions with C—H distances ranging from 0.95 to 1.00 Å and included in the refinement in a riding-model approximation with *U*_iso_(H) = 1.2*U*_eq_(C) or *U*_iso_(H) = 1.5*U*_eq_(C) for methyl H atoms.

### Crystal data

**Table:**

C_16_H_26_O_6_	*F*(000) = 680
*M**_r_* = 314.37	*D*_x_ = 1.245 Mg m^−^^3^
Monoclinic, *P*2_1_/*c*	Mo *K*α radiation, λ = 0.71073 Å
Hall symbol: -P 2ybc	Cell parameters from 9313 reflections
*a* = 12.6548 (11) Å	θ = 2.7–25.0°
*b* = 5.8862 (6) Å	µ = 0.09 mm^−^^1^
*c* = 23.126 (2) Å	*T* = 150 K
β = 103.147 (5)°	Plate, colourless
*V* = 1677.5 (3) Å^3^	0.10 × 0.09 × 0.02 mm
*Z* = 4	

### Data collection

**Table:**

Nonius KappaCCD diffractometer	2893 independent reflections
Radiation source: fine-focus sealed tube	1407 reflections with *I* > 2σ(*I*)
graphite	*R*_int_ = 0.101
Detector resolution: 9 pixels mm^-1^	θ_max_ = 25.0°, θ_min_ = 2.7°
φ scans and ω scans with κ offsets	*h* = −15→15
Absorption correction: multi-scan (*SORTAV*; Blessing 1995)	*k* = −6→6
*T*_min_ = 0.865, *T*_max_ = 1.00	*l* = −27→27
9313 measured reflections	

### Refinement

**Table:**

Refinement on *F*^2^	Primary atom site location: structure-invariant direct methods
Least-squares matrix: full	Secondary atom site location: difference Fourier map
*R*[*F*^2^ > 2σ(*F*^2^)] = 0.068	Hydrogen site location: inferred from neighbouring sites
*wR*(*F*^2^) = 0.191	H-atom parameters constrained
*S* = 1.00	*w* = 1/[σ^2^(*F*_o_^2^) + (0.0826*P*)^2^] where *P* = (*F*_o_^2^ + 2*F*_c_^2^)/3
2893 reflections	(Δ/σ)_max_ < 0.001
205 parameters	Δρ_max_ = 0.24 e Å^−^^3^
0 restraints	Δρ_min_ = −0.26 e Å^−^^3^

### Special details

**Table:**

Geometry. All e.s.d.'s (except the e.s.d. in the dihedral angle between two l.s. planes) are estimated using the full covariance matrix. The cell e.s.d.'s are taken into account individually in the estimation of e.s.d.'s in distances, angles and torsion angles; correlations between e.s.d.'s in cell parameters are only used when they are defined by crystal symmetry. An approximate (isotropic) treatment of cell e.s.d.'s is used for estimating e.s.d.'s involving l.s. planes.
Refinement. Refinement of *F*^2^ against ALL reflections. The weighted *R*-factor *wR* and goodness of fit *S* are based on *F*^2^, conventional *R*-factors *R* are based on *F*, with *F* set to zero for negative *F*^2^. The threshold expression of *F*^2^ > σ(*F*^2^) is used only for calculating *R*-factors(gt) *etc*. and is not relevant to the choice of reflections for refinement. *R*-factors based on *F*^2^ are statistically about twice as large as those based on *F*, and *R*- factors based on ALL data will be even larger.

### Fractional atomic coordinates and isotropic or equivalent isotropic displacement parameters (Å^2^)

**Table:**

	*x*	*y*	*z*	*U*_iso_*/*U*_eq_	
O1	0.3871 (2)	−0.2647 (5)	0.54229 (13)	0.0663 (10)	
O2	0.28611 (18)	−0.5049 (4)	0.48553 (11)	0.0489 (7)	
O3	0.2107 (2)	−0.2869 (4)	0.54593 (11)	0.0503 (7)	
O4	0.62164 (19)	0.3112 (4)	0.70717 (12)	0.0558 (8)	
O5	0.72570 (17)	0.5487 (4)	0.76245 (10)	0.0448 (7)	
O6	0.80169 (19)	0.2813 (4)	0.71320 (11)	0.0460 (7)	
C1	0.4089 (3)	−0.0972 (7)	0.59017 (18)	0.0585 (12)	
H1A	0.3386	−0.0373	0.5968	0.070*	
C2	0.4714 (4)	0.0915 (7)	0.57122 (18)	0.0634 (13)	
H2A	0.4502	0.1443	0.5314	0.076*	
C3	0.5543 (4)	0.1881 (6)	0.60687 (19)	0.0620 (12)	
H3A	0.5873	0.3142	0.5924	0.074*	
C4	0.5991 (3)	0.1123 (6)	0.66812 (17)	0.0494 (11)	
H4A	0.6678	0.0259	0.6699	0.059*	
C5	0.5203 (3)	−0.0339 (7)	0.69112 (17)	0.0589 (12)	
H5A	0.5584	−0.1110	0.7280	0.071*	
H5B	0.4624	0.0625	0.7007	0.071*	
C6	0.4707 (4)	−0.2079 (7)	0.64578 (17)	0.0627 (12)	
H6A	0.5287	−0.3047	0.6365	0.075*	
H6B	0.4213	−0.3065	0.6622	0.075*	
C7	0.2865 (3)	−0.3486 (6)	0.52697 (16)	0.0449 (10)	
C8	0.1825 (3)	−0.6087 (6)	0.45306 (15)	0.0405 (9)	
C9	0.1263 (3)	−0.7330 (6)	0.49534 (16)	0.0555 (12)	
H9A	0.1044	−0.6234	0.5223	0.083*	
H9B	0.1764	−0.8441	0.5185	0.083*	
H9C	0.0620	−0.8117	0.4725	0.083*	
C10	0.1114 (3)	−0.4266 (6)	0.41818 (16)	0.0475 (10)	
H10A	0.0908	−0.3174	0.4457	0.071*	
H10B	0.0459	−0.4964	0.3938	0.071*	
H10C	0.1513	−0.3480	0.3924	0.071*	
C11	0.2218 (3)	−0.7736 (6)	0.41206 (17)	0.0551 (12)	
H11A	0.2735	−0.8804	0.4357	0.083*	
H11B	0.2575	−0.6891	0.3853	0.083*	
H11C	0.1598	−0.8577	0.3886	0.083*	
C12	0.7258 (3)	0.3718 (6)	0.72638 (15)	0.0387 (9)	
C13	0.8304 (3)	0.6424 (6)	0.79714 (15)	0.0389 (9)	
C14	0.8905 (3)	0.4608 (6)	0.83795 (16)	0.0506 (11)	
H14A	0.9138	0.3407	0.8142	0.076*	
H14B	0.8425	0.3961	0.8614	0.076*	
H14C	0.9543	0.5278	0.8646	0.076*	
C15	0.8964 (3)	0.7397 (6)	0.75658 (16)	0.0441 (10)	
H15A	0.8539	0.8562	0.7312	0.066*	
H15B	0.9155	0.6184	0.7318	0.066*	
H15C	0.9628	0.8081	0.7804	0.066*	
C16	0.7894 (3)	0.8280 (6)	0.83263 (16)	0.0498 (10)	
H16A	0.7443	0.9354	0.8053	0.075*	
H16B	0.8513	0.9086	0.8572	0.075*	
H16C	0.7461	0.7591	0.8582	0.075*	

### Atomic displacement parameters (Å^2^)

**Table:**

	*U*^11^	*U*^22^	*U*^33^	*U*^12^	*U*^13^	*U*^23^
O1	0.0400 (15)	0.080 (2)	0.074 (2)	−0.0034 (15)	0.0022 (14)	−0.0451 (17)
O2	0.0407 (14)	0.0519 (16)	0.0495 (16)	−0.0001 (12)	0.0006 (12)	−0.0191 (13)
O3	0.0462 (15)	0.0593 (18)	0.0468 (18)	−0.0065 (13)	0.0135 (14)	−0.0159 (13)
O4	0.0369 (14)	0.0573 (17)	0.071 (2)	−0.0031 (13)	0.0078 (13)	−0.0341 (15)
O5	0.0377 (14)	0.0478 (15)	0.0467 (16)	−0.0061 (12)	0.0049 (12)	−0.0142 (13)
O6	0.0397 (14)	0.0440 (15)	0.0536 (18)	0.0009 (12)	0.0090 (13)	−0.0072 (13)
C1	0.043 (2)	0.067 (3)	0.060 (3)	−0.002 (2)	0.000 (2)	−0.034 (2)
C2	0.090 (3)	0.047 (3)	0.043 (3)	0.003 (2)	−0.005 (2)	−0.002 (2)
C3	0.098 (3)	0.039 (2)	0.054 (3)	−0.014 (2)	0.027 (3)	−0.012 (2)
C4	0.043 (2)	0.052 (2)	0.053 (3)	−0.0016 (19)	0.0120 (19)	−0.022 (2)
C5	0.073 (3)	0.052 (2)	0.048 (3)	−0.011 (2)	0.006 (2)	0.003 (2)
C6	0.086 (3)	0.057 (3)	0.048 (3)	−0.028 (2)	0.022 (2)	−0.009 (2)
C7	0.050 (2)	0.045 (2)	0.035 (2)	0.001 (2)	0.000 (2)	−0.0070 (19)
C8	0.041 (2)	0.041 (2)	0.035 (2)	−0.0049 (17)	−0.0024 (17)	−0.0031 (17)
C9	0.065 (3)	0.052 (3)	0.045 (3)	−0.007 (2)	0.003 (2)	0.001 (2)
C10	0.048 (2)	0.051 (2)	0.040 (2)	−0.0012 (19)	0.0032 (18)	0.0025 (19)
C11	0.055 (2)	0.057 (3)	0.048 (3)	0.003 (2)	0.001 (2)	−0.015 (2)
C12	0.039 (2)	0.042 (2)	0.034 (2)	−0.0034 (19)	0.0052 (18)	0.0001 (18)
C13	0.038 (2)	0.039 (2)	0.035 (2)	−0.0065 (17)	0.0003 (17)	−0.0009 (17)
C14	0.060 (2)	0.044 (2)	0.043 (2)	−0.006 (2)	0.0008 (19)	0.0045 (19)
C15	0.045 (2)	0.042 (2)	0.043 (2)	−0.0025 (18)	0.0069 (18)	−0.0016 (18)
C16	0.052 (2)	0.051 (2)	0.043 (2)	−0.010 (2)	0.0044 (19)	−0.0085 (19)

### Geometric parameters (Å, °)

**Table:**

O1—C7	1.337 (4)	C8—C10	1.510 (4)
O1—C1	1.461 (4)	C8—C11	1.518 (5)
O2—C7	1.328 (4)	C8—C9	1.521 (5)
O2—C8	1.486 (4)	C9—H9A	0.9800
O3—C7	1.197 (4)	C9—H9B	0.9800
O4—C12	1.340 (4)	C9—H9C	0.9800
O4—C4	1.466 (4)	C10—H10A	0.9800
O5—C12	1.334 (4)	C10—H10B	0.9800
O5—C13	1.490 (4)	C10—H10C	0.9800
O6—C12	1.197 (4)	C11—H11A	0.9800
C1—C2	1.486 (6)	C11—H11B	0.9800
C1—C6	1.494 (5)	C11—H11C	0.9800
C1—H1A	1.0000	C13—C15	1.504 (5)
C2—C3	1.307 (5)	C13—C14	1.512 (4)
C2—H2A	0.9500	C13—C16	1.527 (5)
C3—C4	1.470 (5)	C14—H14A	0.9800
C3—H3A	0.9500	C14—H14B	0.9800
C4—C5	1.503 (5)	C14—H14C	0.9800
C4—H4A	1.0000	C15—H15A	0.9800
C5—C6	1.497 (5)	C15—H15B	0.9800
C5—H5A	0.9900	C15—H15C	0.9800
C5—H5B	0.9900	C16—H16A	0.9800
C6—H6A	0.9900	C16—H16B	0.9800
C6—H6B	0.9900	C16—H16C	0.9800
			
C7—O1—C1	117.0 (3)	C8—C9—H9B	109.5
C7—O2—C8	120.5 (3)	H9A—C9—H9B	109.5
C12—O4—C4	117.1 (3)	C8—C9—H9C	109.5
C12—O5—C13	119.9 (3)	H9A—C9—H9C	109.5
O1—C1—C2	107.6 (3)	H9B—C9—H9C	109.5
O1—C1—C6	109.2 (3)	C8—C10—H10A	109.5
C2—C1—C6	111.7 (3)	C8—C10—H10B	109.5
O1—C1—H1A	109.4	H10A—C10—H10B	109.5
C2—C1—H1A	109.4	C8—C10—H10C	109.5
C6—C1—H1A	109.4	H10A—C10—H10C	109.5
C3—C2—C1	122.9 (4)	H10B—C10—H10C	109.5
C3—C2—H2A	118.5	C8—C11—H11A	109.5
C1—C2—H2A	118.5	C8—C11—H11B	109.5
C2—C3—C4	123.6 (4)	H11A—C11—H11B	109.5
C2—C3—H3A	118.2	C8—C11—H11C	109.5
C4—C3—H3A	118.2	H11A—C11—H11C	109.5
O4—C4—C3	109.2 (3)	H11B—C11—H11C	109.5
O4—C4—C5	106.9 (3)	O6—C12—O5	128.4 (3)
C3—C4—C5	111.9 (3)	O6—C12—O4	125.7 (3)
O4—C4—H4A	109.6	O5—C12—O4	106.0 (3)
C3—C4—H4A	109.6	O5—C13—C15	110.9 (3)
C5—C4—H4A	109.6	O5—C13—C14	109.5 (3)
C6—C5—C4	110.5 (3)	C15—C13—C14	112.8 (3)
C6—C5—H5A	109.6	O5—C13—C16	100.6 (3)
C4—C5—H5A	109.6	C15—C13—C16	111.6 (3)
C6—C5—H5B	109.6	C14—C13—C16	110.8 (3)
C4—C5—H5B	109.6	C13—C14—H14A	109.5
H5A—C5—H5B	108.1	C13—C14—H14B	109.5
C1—C6—C5	111.0 (3)	H14A—C14—H14B	109.5
C1—C6—H6A	109.4	C13—C14—H14C	109.5
C5—C6—H6A	109.4	H14A—C14—H14C	109.5
C1—C6—H6B	109.4	H14B—C14—H14C	109.5
C5—C6—H6B	109.4	C13—C15—H15A	109.5
H6A—C6—H6B	108.0	C13—C15—H15B	109.5
O3—C7—O2	127.1 (3)	H15A—C15—H15B	109.5
O3—C7—O1	125.8 (3)	C13—C15—H15C	109.5
O2—C7—O1	107.1 (3)	H15A—C15—H15C	109.5
O2—C8—C10	109.2 (3)	H15B—C15—H15C	109.5
O2—C8—C11	101.6 (3)	C13—C16—H16A	109.5
C10—C8—C11	111.1 (3)	C13—C16—H16B	109.5
O2—C8—C9	111.1 (3)	H16A—C16—H16B	109.5
C10—C8—C9	112.1 (3)	C13—C16—H16C	109.5
C11—C8—C9	111.2 (3)	H16A—C16—H16C	109.5
C8—C9—H9A	109.5	H16B—C16—H16C	109.5
